# Health-related quality of life in women and men with long COVID

**DOI:** 10.1371/journal.pone.0357749

**Published:** 2026-10-26

**Authors:** Sara Garces-Arilla, Camino Fidalgo, Magdalena Mendez-Lopez, Vanesa Hidalgo, Esther del Corral-Beamonte, Jorge Osma

**Affiliations:** 1 Department of Psychology and Sociology, University of Zaragoza, Zaragoza, Spain; 2 IIS Aragon, Zaragoza, Spain; 3 Laboratory of Social Cognitive Neuroscience, Department of Psychobiology, IDOCAL, University of Valencia, Valencia, Spain; 4 Villanova Royo Hospital, Zaragoza, Spain; Ruhr University Bochum, GERMANY

## Abstract

Long COVID is associated with persistent physical and emotional symptoms that may substantially impair health-related quality of life, with growing evidence suggesting that these outcomes differ between women and men. This cross-sectional study examined sex differences in health-related quality of life, lifestyle habits, and symptom severity in long COVID, and explored factors associated with health-related quality of life in women and men. A total of 342 adults with long COVID (mean age = 51.20 years, SD = 10.49; 265 women and 77 men) completed a battery of questionnaires during a single, face-to-face individual assessment session. The questionnaires assessed health-related quality of life, smoking behaviour, physical activity, dyspnoea, fatigue, and anxiety and depressive symptoms. Controlling for age and months since diagnosis, women reported lower quality of life and greater dyspnoea severity than men, whereas no sex differences were observed in smoking behaviour, fatigue severity, or anxiety and depressive symptoms. In sex-stratified exploratory analyses, poorer quality of life was associated with greater dyspnoea, fatigue, and emotional symptom severity in women, whereas in men it was associated with greater fatigue. However, interactions between sex and these factors were not statistically significant. These findings indicate sex differences in health-related quality of life and dyspnoea severity among individuals with long COVID. Larger, adequately powered studies are needed to determine whether the factors associated with health-related quality of life differ by sex.

## 1. Introduction

Long COVID is defined as a condition in which patients continue to experience or develop new symptoms of COVID-19 three months after contracting COVID-19 [1]. Approximately 10% of individuals with prior COVID-19 infection experience long COVID, affecting more than 65 million individuals across the world [2]. The most common symptoms include somatic manifestations (e.g., fatigue, shortness of breath, olfactory and taste disorders), cognitive difficulties (such as attention and memory alterations), and emotional disturbances (including depression and anxiety) [3–5]. Emerging evidence suggests that long COVID does not affect all population groups equally, with women appearing to be at higher risk of developing several long COVID symptoms, particularly fatigue, headache, cognitive complaints, and mood disturbances [6]. Overall, these symptoms can persist for months and often interfere with daily functioning, work activities, and overall quality of life [7,8]. Recent evidence further suggests that perceived physical health in long COVID is closely linked to overall symptom severity, which remains strongly associated with health perceptions after accounting for sex and behavioural factors [9]. Consequently, quality of life is now considered an essential indicator for assessing the impact of long COVID on individuals’ functional status and overall well-being.

Quality of life is a multidimensional concept that refers to an individual’s overall perception of their well-being, influenced by their health, psychological state, level of independence, social relationships, and environment [10]. In the context of long COVID, the assessment of health-related quality of life (HRQoL), a measure of perceived physical and emotional functioning, has therefore become a central outcome of interest.

To properly assess quality of life, instruments must be designed to encompass its inherently subjective and multidimensional components. However, during the first phases of the pandemic no COVID-19–specific instruments were available to evaluate HRQoL in this population. Therefore, researchers had to rely on generic questionnaires or ad hoc symptom checklists [11]. Among the most frequently used validated measures of HRQoL in long COVID are the 36-Item Short Form Survey (SF-36; [12]), which assesses physical and mental health functioning (e.g., [13]), and the EuroQol 5-Dimension questionnaire (EQ-5D; [14]), one of the most widely used generic preference-based instruments internationally, with extensive adoption across countries and health conditions (e.g., [15]).

Several studies suggest that women experience a greater decline in HRQoL after COVID-19, with lower scores across multiple domains and female sex emerging as an independent predictor of reduced EQ-5D outcomes [16,17]. Consistently, a systematic review highlighted female sex as one of the most frequent factors associated with poorer post-COVID quality of life [18]. Large-scale syntheses of EQ-5D data further indicate that sex is a relevant determinant of post-COVID HRQoL, with women generally reporting lower health status than men across multiple cohorts [19]. Several mechanisms may contribute to this increased vulnerability: women may exhibit a more pronounced inflammatory response, which could prolong post-COVID symptomatology [6]. Women also show a higher prevalence of anxiety, depression, and other mental health symptoms associated with reduced HRQoL [17].

In addition to sex-related differences in HRQoL, other factors may also be related to post-COVID health outcomes. Several studies have explored behavioural factors associated with post-COVID outcomes. Although evidence remains limited, longitudinal findings indicate that smoking prior to the infection may predispose individuals to a more impaired functional recovery [20], suggesting that pre-infection smoking may contribute to a more severe functional burden during the post-acute phase. Importantly, this pattern extends to the post-infection period, where continued smoking has also been associated with adverse long-term outcomes. A two-year prospective cohort study found that current smokers exhibited more than threefold higher odds of persistent long COVID symptoms and widespread symptomatology [21]. The study also identified female sex as an independent predictor of long COVID, although it did not evaluate sex-specific associations with smoking. While sex-related differences in long COVID remain insufficiently explored, smoking behaviour is known to vary by sex, with hospital-based cohorts reporting higher smoking rates among men [22]. Whether the consequences of smoking on long COVID differ between men and women therefore remains unclear.

Reduced physical activity is also a widely reported feature of long COVID and has been consistently linked to poorer HRQoL, with studies documenting post-infection declines associated with persistent symptoms such as fatigue and dyspnoea [23,24] and with post-exertional malaise frequently acting as a barrier to activity tolerance [25]. Tak [25] furthermore observed that individuals with reduced activity tolerance reported lower EQ-5D utility values, underscoring the relevance of physical functioning for HRQoL in long COVID. In line with this, evidence indicates that physical activity is positively associated with perceived physical health in long COVID independently of sex, although sex-specific analyses of physical activity patterns remain scarce [9]. Considering sex, longitudinal analyses show that female sex is associated with distinct EQ-5D trajectories over time, with women exhibiting lower HRQoL scores in association with fatigue and functional limitations [26]. Taken together, these findings highlight the importance of considering physical activity patterns, and their potential variation by sex, when evaluating long COVID outcomes.

Overall, given that lifestyle habits, as well as physical and emotional symptoms, may differ between women and men with long COVID and may show different associations with HRQoL differently by sex, the present study had three main objectives. First, we conducted a preliminary examination of sex differences in long COVID symptomatology, HRQoL scores, smoking behaviour, physical activity, dyspnoea and fatigue severity, and anxiety and depressive symptoms. Second, we examined the exploratory associations between smoking status, physical activity, dyspnoea, fatigue, and anxiety and depressive symptoms to HRQoL scores, analysed separately for women and men. Third, we conducted an exploratory analysis to assess whether sex moderated the associations between these factors and HRQoL.

Based on previous evidence, we hypothesised that women with long COVID would report lower HRQoL and greater symptom severity than men, particularly in terms of dyspnoea and emotional symptomatology [6] (H1). In light of evidence linking fatigue and functional limitations to reduced activity tolerance in long COVID, we hypothesised that women would report lower levels of physical activity than men [25,26] (H1). We further hypothesised that greater dyspnoea severity, greater fatigue severity, and higher levels of anxiety and depressive symptoms would be associated with poorer HRQoL [8,9,17] (H2). Finally, based on previous evidence of sex-related differences in symptom burden and HRQoL [6,17], we explored whether sex moderated the associations between these factors and HRQoL.

## 2. Materials and methods

### 2.1. Participants

This cross-sectional study included participants from the ARACOV project, which aimed to characterise patients with long COVID and to analyse the impact of a nutritional and rehabilitation intervention on their quality of life. Participants were recruited through the Primary Care and Occupational Health services participating in the ARACOV project. According to the Aragón regional clinical protocol [27], when a patient was diagnosed with long COVID, the healthcare professional informed the patient about the ARACOV cohort and offered them the opportunity to participate. Patients who were interested contacted the research team by email or telephone and were scheduled for a baseline assessment. During this visit, eligibility was verified according to the predefined inclusion and exclusion criteria, written informed consent was obtained, and patients who met the eligibility criteria were included in the study.

Long COVID was diagnosed by a primary care or occupational physician as part of the assessment performed by the Primary Care team, according to the Aragón regional clinical protocol [27]. The diagnosis required persistence of symptoms attributable to SARS-CoV-2 infection for more than 12 weeks after the acute phase that could not be explained by an alternative diagnosis. The diagnostic assessment included a review of medical history and comorbidities, physical examination, assessment of persistent symptoms, and complementary investigations aimed at identifying organ damage and excluding alternative diagnoses, including blood tests, electrocardiography (ECG), chest radiography (with chest computed tomography when indicated), and respiratory functional assessment (spirometry and/or the 6-minute walk test or the 1-minute sit-to-stand test), when clinically appropriate.

The inclusion criteria were: providing written informed consent; being aged ≥ 18 years; having a documented SARS-CoV-2 infection in the clinical record, confirmed by PCR, antigen testing, or serology; having the ability to understand the study questionnaires; and being able to read in Spanish. The exclusion criteria were: the presence of symptoms that preceded the acute SARS-CoV-2 infection; symptoms attributable to organ-specific damage caused by severe COVID-19; having severe neurological disease; presenting respiratory insufficiency, rheumatic pathology, or an acute musculoskeletal injury; having received immunosuppressants or corticosteroids in the previous weeks; and current participation in a clinical trial.

As shown in Fig 1, in a first attempt 347 participants were assessed for eligibility. Of these, 5 participants were excluded for the following reasons: three did not meet the eligibility criteria because their symptoms had been present before the acute SARS-CoV-2 infection, one was participating in a clinical trial, and one had a medical or psychological condition that, in the investigator’s opinion, could interfere with compliance with the study procedures. All excluded participants were women. The final study sample therefore included all eligible participants recruited during the study period.

**Fig 1:**
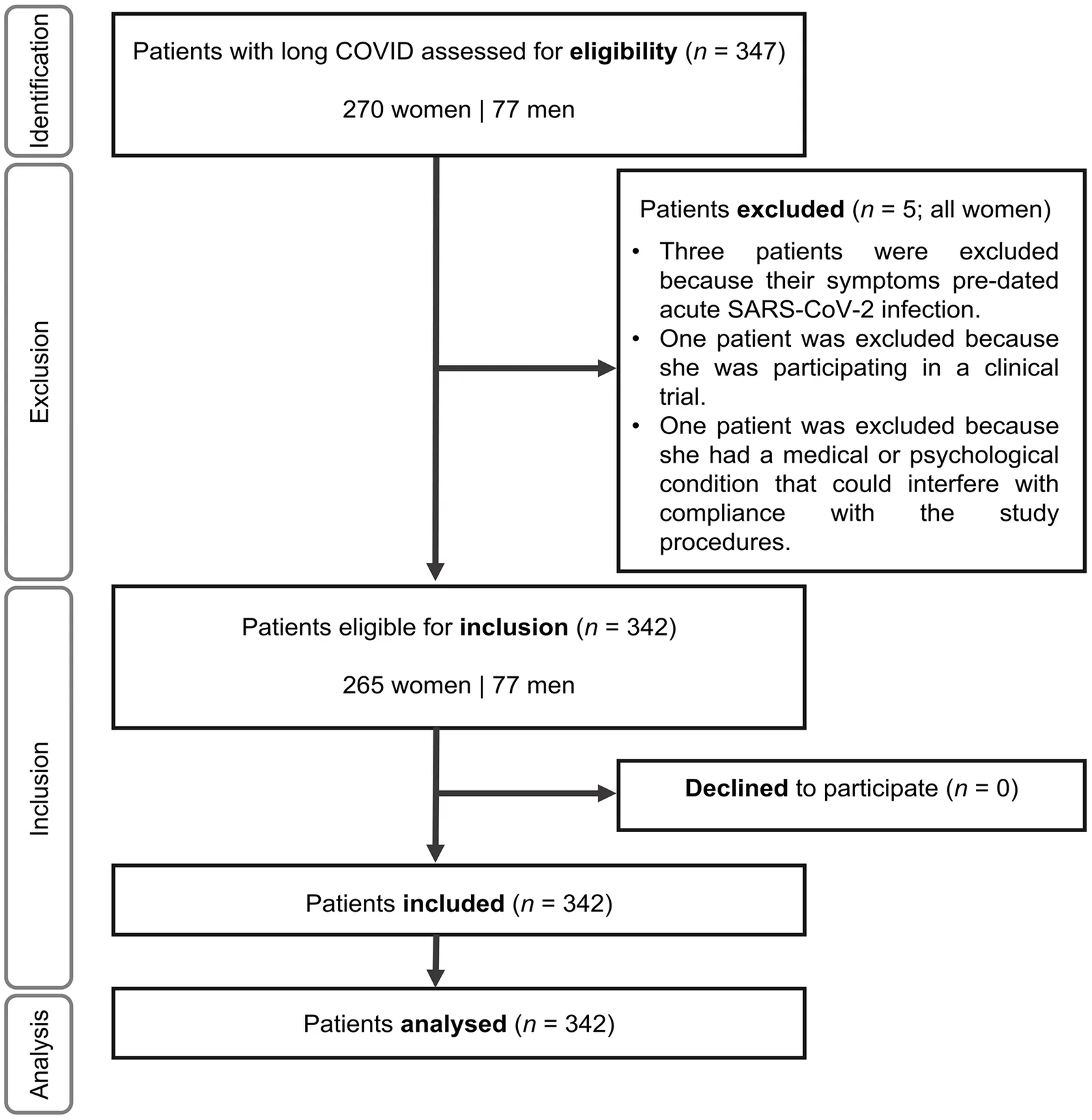
Flow diagram of participant selection and inclusion in the study.

The final sample comprised 342 participants diagnosed with long COVID (77.5% female, *n* = 265), aged between 18 and 87 years (*M* = 51.20, *SD* = 10.49). The time interval between diagnosis and assessment ranged from approximately 2–40 months (*M* = 22.16, *SD* = 9.59). The most commonly reported long COVID symptoms related to physical functioning in this sample were chronic fatigue (84.2%) and dyspnoea (70.5%). Table 1 summarises the characteristics of the sample and the sex-based differences.

**Table 1:** Descriptive characteristics and sex-based differences in long COVID symptomatology.

	Total (*n* = 342)	Women (*n* = 265)	Men (*n* = 77)	*p*
Age, *M* (*SD*)	51.20 (10.49)	50.55 (10.38)	53.47 (10.26)	.031*
Months^a^, *M* (*SD*)	22.16 (9.59)	21.92 (9.41)	22.98 (10.20)	.395
**Living arrangement**, *n* (%)				.501
Living alone	48 (14.0)	39 (14.7)	9 (11.7)	
Living with others	294 (86.0)	226 (85.3)	68 (88.3)	
**Symptomatology**, *n* (%)				
*Respiratory symptoms*				
Dyspnoea	241 (70.5)	193 (72.8)	48 (62.3)	.076
Cough	103 (30.1)	83 (31.3)	20 (26.0)	.368
*Cardiovascular symptoms*				
Chest pain	82 (24.0)	63 (23.8)	19 (24.7)	.870
Chest tightness	78 (22.8)	64 (24.2)	14 (18.2)	.272
Palpitations	131 (38.3)	114 (43.0)	17 (22.1)	<.001**
*Neurological symptoms*				
Headache	189 (55.3)	159 (60.0)	30 (39.0)	.001**
Dizziness	130 (38.0)	103 (38.9)	27 (35.1)	.545
Tinnitus	65 (19.0)	50 (18.9)	15 (19.5)	.904
Ageusia/ Anosmia	81 (23.7)	67 (25.3)	14 (18.2)	.197
Sleep disturbances	170 (49.7)	137 (51.7)	33 (42.9)	.172
Paraesthesia	144 (42.1)	112 (42.3)	32 (41.6)	.912
Myalgia	218 (63.7)	175 (66.0)	43 (55.8)	.101
Brain fog	252 (73.7)	204 (77.0)	48 (62.3)	.010*
Memory problems	252 (73.7)	204 (77.0)	48 (62.3)	.010*
Concentration problems	220 (64.3)	182 (68.7)	38 (49.4)	.002*
*Gastrointestinal symptoms*				
Abdominal pain	63 (18.4)	55 (20.8)	8 (10.4)	.039*
Nausea	57 (16.7)	49 (18.5)	8 (10.4)	.093
Diarrhoea	77 (22.5)	67 (25.3)	10 (13.0)	.023*
Anorexia	10 (2.9)	8 (3.0)	2 (2.6)	.847
*Systemic symptoms*				
Chronic fatigue	288 (84.2)	226 (85.3)	62 (80.5)	.313
Fever	21 (6.1)	18 (6.8)	3 (3.9)	.351
General pain	133 (38.9)	107 (40.4)	26 (33.8)	.295
Arthralgia	187 (54.7)	153 (57.7)	34 (44.2)	.035*
Ear or throat pain	71 (20.8)	57 (21.5)	14 (18.2)	.526

*Note*. ^a^ Months since diagnosis; ** *p* ≤ .001; *p < .05.

The study was designed in accordance with the fundamental ethical principles outlined in the Declaration of Helsinki and with the standards of Good Clinical Practice. This study and all its procedures were approved by the Research Ethics Committee of the Autonomous Community of Aragón (CEICA) (reference number: PI22/334). In addition, the study complies with Spanish Law 41/2002 of 14 November on patient autonomy and rights and obligations regarding clinical information and documentation, with Regulation (EU) 2016/679 of the European Parliament and of the Council of 27 April 2016 (GDPR), and with Organic Law 3/2018 of 5 December on the Protection of Personal Data and the Guarantee of Digital Rights. The participants were recruited from September 21, 2022 to September 27, 2023. Written informed consent was obtained from all participants prior to data collection.

### 2.2. Measurements and procedure

Participants who met the inclusion criteria were provided with a study information sheet and asked to provide written informed consent in a face-to-face setting with nurses involved in the ARACOV project at the following healthcare centres in Aragón: Lozano Blesa University Hospital; Huesca Pirineos Health Centre; and Teruel Ensanche Health Centre. During the same session, data were collected on sociodemographic characteristics (age, sex, living arrangement, and date of long COVID diagnosis), long COVID symptomatology, HRQoL, smoking behaviour and physical activity, dyspnoea and fatigue severity and emotional symptomatology.

#### 2.2.1. Long COVID symptomatology.

Long COVID symptoms reported by patients were assessed using an ad hoc symptom checklist developed for the present study. For each symptom, participants indicated whether it was present or absent using a dichotomous response format (Yes/No). Symptoms were organised into five domains: respiratory (dyspnoea and cough); cardiovascular (chest pain, chest tightness and palpitations); neurological (headache, dizziness, tinnitus, loss of taste or smell, sleep disturbances, paraesthesia, myalgia, brain fog, memory problems and difficulty concentrating); gastrointestinal (abdominal pain, nausea, diarrhoea and loss of appetite); and systemic (chronic fatigue, fever, general pain, arthralgia and ear or throat pain).

#### 2.2.2. Health-related quality of life.

The study assessed HRQoL using the Visual Analogue Scale of the EQ-5D [14], Spanish validation by [28], as used in previous research (e.g., [29]). The EQ-5D can be applied to both general population and patient groups with various medical conditions. Participants rated their current health on a 0–100 scale, where 0 represents the worst imaginable health state and 100 the best.

#### 2.2.3. Smoking and physical activity.

Smoking behaviour was assessed by asking participants to indicate their smoking status (current smoker, ex-smoker or never smoker) and, for those who smoked, their smoking exposure in pack-years.

Physical activity was measured using a single item assessing the number of weekly hours devoted specifically to physical exercise. Housework, childcare and other domestic activities were not included in this item.

#### 2.2.4. Dyspnoea and fatigue severity.

Dyspnoea severity was assessed using the modified Medical Research Council (mMRC) scale [30]. The mMRC is a single-item measure in which participants select the one grade that best describes their level of breathlessness. The scale classifies dyspnoea into five grades: Grade 0 (dyspnoea only with strenuous exercise), Grade 1 (short of breath when hurrying on level ground or walking up a slight hill), Grade 2 (walks slower than people of the same age due to breathlessness or has to stop when walking at their own pace), Grade 3 (stops for breath after walking about 100 metres or after a few minutes on level ground), and Grade 4 (too breathless to leave the house or breathless when dressing or undressing).

Fatigue severity was assessed using the Fatigue Severity Scale (FSS; [31] Spanish validation by [32]). The FSS is a self-administered questionnaire composed of nine items that relate fatigue severity to daily activities and is commonly used to evaluate fatigue associated with medical conditions or ageing. Each item is rated on a seven-point scale from 1 (strongly disagree) to 7 (strongly agree). The scale showed excellent internal consistency in the present sample (Cronbach’s α = .93).

#### 2.2.5. Emotional symptomatology.

Anxious and depressive symptomatology experienced over the past week were assessed using the Overall Anxiety Severity and Impairment Scale (OASIS; [33] Spanish validation by [34]) and the Overall Depression Severity and Impairment Scale (ODSIS; [35] Spanish validation by [34]), respectively. These questionnaires evaluate the frequency, intensity, and daily-life interference of emotional symptoms. Both instruments comprise five items rated on a five-point Likert scale ranging from 0 (little/not at all) to 4 (extreme), with higher scores indicating greater symptom severity and impairment. Internal consistency was excellent in the present sample (OASIS: Cronbach’s α = .93; ODSIS: Cronbach’s α = .96).

### 2.3. Statistical analysis

The final dataset included the full sample of 342 eligible participants. The proportion of missing data was low, ranging from 1.17% to 4.39% across the included variables (see Table 2). SPSS used listwise deletion to exclude cases with missing values in any of the variables included in each analysis.

**Table 2:** Mean (M) and standard deviation (SD) for HRQoL, smoking behaviour, physical activity, dyspnoea, fatigue and emotional symptomatology.

Variables	Total (*n* = 342)	Women (*n* = 265)	Men (*n* = 77)	Missing data^d^, *n* (%)
HRQoL^a^, *M* (*SD*)	52.77 (19.39)	51.86 (19.45)	56.10 (18.93)	15 (4.4%)
Tobacco-related habits				
Smoking status, *n* (%)				4 (1.2%)
Smoker, *n* (%)	40 (11.7%)	32 (12.1%)	8 (10.4%)	
Ex-smoker, *n* (%)	137 (40.1%)	98 (37.0%)	39 (50.6%)	
Never smoker, *n* (%)	161 (47.1%)	132 (49.8%)	29 (37.7%)	
Pack-years^b^, *M* (*SD*)	14.13 (51.76)	15.23 (54.20)	10.34 (42.38)	–
Physical activity^c^, *M* (*SD*)	3.38 (3.65)	3.15 (3.43)	4.16 (4.26)	8 (2.3)
Dyspnoea, *M* (*SD*)	2.72 (.86)	2.78 (.84)	2.52 (.93)	6 (1.8)
Fatigue, *M* (*SD*)	5.77 (1.39)	5.81 (1.35)	5.63 (1.51)	4 (1.2)
Anxiety, *M* (*SD*)	7.59 (5.12)	7.82 (5.09)	6.83 (5.21)	5 (1.5)
Depression, *M* (*SD*)	6.39 (5.51)	6.54 (5.61)	5.87 (5.17)	5 (1.5)

*Note*. ^a^ HRQoL = Health-related quality of life; ^b^ Smoking exposure expressed as pack-years; ^c^ number of hours of physical activity per week; ^d^ Percentages of missing data were calculated based on the full sample of 342 participants.

Independent-samples *t*-tests were conducted to assess sex differences in age, months since diagnosis, and smoking exposure expressed as pack-years. Age differed significantly between men and women (*p* = .031), and it was therefore included as a covariate in subsequent analyses due to both this between-sex difference and its wide range (18–87 years). Likewise, months since diagnosis was included as a covariate, as participants differed substantially in the duration of their condition (2–40 months) and its potential influence on the study variables.

Chi-square tests (*X*^2^) were used to analyse sex differences in living arrangement, long COVID symptomatology and in smoking status (current smoker, ex-smoker, or never smoker), as these variables were categorical.

Univariate analyses of covariance (ANCOVAs) were conducted to examine sex differences in HRQoL, weekly hours of physical activity, fatigue severity, and emotional symptomatology, adjusting for age and months since diagnosis (see Table 3). However, the model examining weekly physical activity violated the assumption of homogeneity of variances, as indicated by Levene’s test (*p* = .020). Therefore, HC3 heteroscedasticity-consistent standard errors were additionally calculated to account for unequal variances.

**Table 3:** Sex differences in HRQoL, physical activity, fatigue, anxiety, depression and dyspnoea, adjusted for age and months since diagnosis.

Criterion	Predictors	Univariate ANCOVAs		
		*F*(df_1_, df_2_)	*η* ^2^_*p*_	*p*
HRQoL				
	Sex	4.00 (1,323)	.012	.046^*^
	Age	.17 (1, 323)	.001	.639
	Months^a^	.75 (1, 323)	.003	.388
Physical activity^b^				
	Sex	4.70 (1,330)	.014	.031^*,c^
	Age	.04 (1,330)	<.001	.833
	Months^a^	.31 (1,330)	.001	.576
Fatigue				
	Sex	.85 (1,334)	.003	.358
	Age	.17 (1,334)	.001	.683
	Months^a^	.61 (1,334)	.002	.434
Anxiety				
	Sex	1.62 (1,333)	.005	.204
	Age	3.07 (1,333)	.009	.081
	Months^a^	.001 (1,333)	<.001	.970
Depression				
	Sex	.51 (1,333)	.002	.477
	Age	2.42 (1,333)	.007	.121
	Months^a^	1.05 (1,333)	.003	.307
		Ordinal regression		
Dyspnoea		Wald (df)	*OR*	*p*
	Sex	8.11 (1)	2.02	.004^*^
	Age	7.53 (1)	1.03	.006^*^
	Months^a^	1.16 (1)	1.01	.281

*Note*. HRQoL = Health-related quality of life; ^a^ Months since diagnosis; ^b^ number of hours of physical activity per week; ^c^ For physical activity, the HC3-robust test of the sex effect yielded a *p* value of.055; ^*^p < .05.

As complementary exploratory analyses, hierarchical multiple linear regressions were conducted separately for women and men to characterise the pattern of associations with HRQoL within each sex. Step 1 included age and months since diagnosis; Step 2 added smoking status; Step 3 added dyspnoea severity; and Step 4 added physical activity, fatigue severity, anxiety, and depressive symptomatology. For conciseness, only the fully adjusted model (Model 4) is presented in Table 4 and Table 5. Smoking status was coded using two dummy variables (smoker and ex-smoker), with never smokers serving as the reference category. Tolerance values ranged from  .37 to  .99 and VIF values from 1.00 to 2.75, indicating no issues of multicollinearity. Bootstrap confidence intervals based on 5,000 resamples were obtained for the coefficients of the male regression model as a sensitivity analysis.

**Table 4:** Factors associated with HRQoL in women.

HRQoL^a^					
Step 4: *R*^2^ = .37, *Adj R*^2^ = .35, *F* change = 18.38, *p* < .001					
Predictors	*B*	*β*	*t*	*p*	95%, *CI*
Age	−.037	−.020	−.37	.709	−.23,.160
Months since diagnosis	−.031	−.015	−.29	.774	−.25,.18
Dummy_smoker^b^	−.098	−.002	−.03	.976	−6.64, 6.44
Dummy_ex-smoker^b^	−.498	−.013	−.23	.820	−4.80, 3.80
Physical activity^c^	.024	.004	.08	.938	−.58,.63
Dyspnoea	−4.893	−.212	−3.51	<.001**	−7.64, −2.15
Fatigue	−2.804	−.196	−3.09	.002*	−4.59, −1.02
Anxiety	−.657	−.172	−2.14	.034*	−1.26, − .05
Depression	−.810	−.238	−3.00	.003*	−1.34, − .28

*Note*. ^a^ HRQoL = Health-related quality of life; ^*b*^ Never-smokers were the reference category; ^c^ Number of hours of physical activity per week; ** *p* ≤ .001; * *p* < .05.

**Table 5:** Factors associated with HRQoL in men.

HRQoL^a^					
Step 4: *R*^2^ = .44, *Adj R*^2^ = .36, *F* change = 6.11, *p* < .001					
Predictors	*B*	*β*	*t*	*p*	95%, *CI*
Age	.228	.125	1.13	.262	−0.17,.63
Months since diagnosis	.392	.213	2.05	.044*	0.01,.77
Dummy_smoker^b^	2.873	.047	.42	.674	−10.70, 16.44
Dummy_ex-smoker^b^	−1.621	−.043	−.38	.704	−10.12, 6.88
Physical activity^c^	.652	.147	1.43	.157	−0.26, 1.56
Dyspnoea	−2.232	−.109	−.93	.354	−7.01, 2.55
Fatigue	−5.377	−.432	−3.71	<.001**	−8.27, −2.48
Anxiety	.272	.074	.48	.634	−0.86, 1.41
Depression	−.646	−.177	−1.15	.255	−1.77,.48

*Note*. ^a^ HRQoL = Health-related quality of life; ^*b*^ Never-smokers were the reference category; ^c^ Number of hours of physical activity per week; ** *p* ≤ .001; * *p* < .05.

To examine whether sex moderated the associations between the examined predictors and HRQoL, an additional hierarchical multiple linear regression analysis was conducted in the full sample. Step 1 included age, months since diagnosis, and sex. Step 2 added smoking status and physical activity; Step 3 added dyspnoea severity and fatigue; and Step 4 added anxiety and depressive symptomatology. In Step 5, seven sex-by-predictor interaction terms were entered simultaneously: sex × current smoking, sex × former smoking, sex × physical activity, sex × dyspnoea, sex × fatigue, sex × anxiety, and sex × depressive symptoms. Continuous predictors included in product terms were mean-centred before the interaction terms were calculated, whereas smoking-status dummies retained their original coding. For transparency, coefficients from both the main-effects model (Model 4) and the final interaction model (Model 5) are presented in Table 6. In the main-effects model, tolerance values ranged from  .40 to  .95 and VIF values ranged from 1.05 to 2.48, indicating no problematic multicollinearity. In the final interaction model, despite mean-centring the continuous predictors before calculating the product terms, collinearity was higher for some anxiety- and depression-related main-effect and interaction terms (tolerance = .08-.93; VIF = 1.08–13.23).

**Table 6:** Factors associated with HRQoL and sex-by-predictor interaction analyses in the full sample.

HRQoL^a^					
Step 4: *R*^2^ = .37, *Adj R*^2^ = .35, F change = 22.53, *p* < .001					
Predictors	*B*	*β*	*t*	*p*	95%, *CI*
Age	.037	.020	.417	.677	−.14,.21
Months since diagnosis	.069	.034	.733	.464	−.12,.25
Sex^b^	−1.869	−.041	−.878	.381	−6.06, 2.32
Dummy_smoker^c^	−.253	−.004	−.086	.931	−6.03, 5.52
Dummy_ex-smoker^c^	.020	.001	.011	.992	−3.75, 3.79
Physical activity^d^	.206	.039	.816	.415	−.29,.70
Dyspnoea	−4.182	−.187	−3.514	<.001^**^	−6.53, −1.84
Fatigue	−3.361	−.242	−4.438	<.001^**^	−4.85, −1.87
Anxiety	−.412	−.109	−1.522	.129	−.95,.12
Depression	−.864	−.249	−3.575	<.001^**^	−1.34, − .39
**Step 5:** ***R***^**2**^ **= .38,** ***Adj R***^**2**^ **= .35,** ***F*** **change = 1.07,** ***p*** **= .384**					
**Predictors**	***B***	***β***	***t***	***p***	**95%,** ***CI***
Age	.021	.011	.231	.817	−.156,.198
Months since diagnosis	.075	.037	.785	.433	−.113,.262
Sex^b^	−2.432	−.053	−.711	.477	−9.161, 4.297
Dummy_smoker^c^	−.000	.000	.000	1.000	−12.955, 12.955
Dummy_ex-smoker^c^	−.407	−.010	−.099	.921	−8.499, 7.686
Physical activity^d^	.760	.142	1.635	.103	−.155, 1.675
Dyspnoea	−1.276	−.057	−.536	.593	−5.963, 3.411
Fatigue	−5.423	−.390	−3.628	<.001**	−8.364, −2.481
Anxiety	.326	.086	.557	.578	−.826, 1.478
Depression	−.850	−.245	−1.487	.138	−1.975,.275
Sex × current smoker	.476	.007	.065	.948	−13.946, 14.898
Sex × former smoker	−.006	.000	−.001	.999	−9.114, 9.101
Sex × physical activity	−.707	−.108	−1.268	.206	−1.804,.390
Sex × dyspnoea	−3.862	−.147	−1.414	.159	−9.238, 1.514
Sex × fatigue	2.747	.168	1.572	.117	−.691, 6.185
Sex × anxiety	−.978	−.224	−1.479	.140	−2.278,.323
Sex × depression	.044	.011	.070	.945	−1.200, 1.288

*Note*. ^a^ HRQoL = Health-related quality of life; ^*b*^ Sex was coded 0 = men and 1 = women; ^c^ Never-smokers were the reference category; ^d^ number of hours of physical activity per week; ** *p* ≤ .001.

All statistical analyses were conducted using IBM SPSS Statistics version 29.

## 3. Results

### 3.1. Exploratory sex differences across variables

#### 3.1.1. Age and long COVID symptomatology.

Age showed a significant difference, with women being younger than men (*p* = .031). Months since diagnosis did not show a significant difference by sex (*p* = .395). Related to long COVID symptomatology, as assessed using the ad hoc symptom checklist, women had a higher prevalence than men of palpitations (*p* < .001), headaches (*p* = .001), brain fog (*p* = .010), memory problems (*p* = .010), concentration problems (*p* = .002), abdominal pain (*p* = .039), diarrhoea (*p* = .023) and arthralgia (*p* = .035). No other symptoms were significant (*p* ≥ .076). See Table 1 for more details.

#### 3.1.2. Health-related quality of life.

Controlling for age and months since diagnosis, sex differences were found in HRQoL scores, *F*(1, 323) = 4.00, *p* = .046, η^2^_*p*_ = .012, with women reporting lower scores than men. Neither covariate showed a significant association (both *p*s ≥ .388). See Table 2 and Table 3.

#### 3.1.3. Smoking and physical activity.

No sex differences were found in smoking status (current smoker, ex-smoker, or never smoker), *X*^2^(2) = 4.80, *p* = .091. Also, no sex differences were observed in smoking exposure expressed as pack-years, *t*(340) = −.729, *p* = .467. See Table 2.

In contrast, after controlling for age and months since diagnosis, women reported lower levels of physical activity than men, *F*(1, 330) = 4.70, *p* = .031, η^2^_*p*_ = .014. However, inference based on heteroscedasticity-consistent standard errors (HC3) indicated that this difference did not reach statistical significance, *B* = 1.04, robust *SE* = 0.54, *t* = 1.92, *p* = .055, 95% *CI* [−.02, 2.10], although the *p*-value was close to the conventional  .05 threshold. Neither age nor months since diagnosis was significantly associated with physical activity in the robust analysis (both *p*s ≥ .585). See Tables 2 and 3.

#### 3.1.4. Dyspnoea and fatigue severity.

Controlling for age and months since diagnosis, significant sex differences were observed in dyspnoea severity, with women having higher odds of reporting higher dyspnoea levels than men (*B* = .70, *SE* = .25, *p* = .004, *OR* = 2.02). Age was also significantly associated with dyspnoea severity, with older participants presenting greater odds of reporting higher dyspnoea severity (*B* = .03, *SE* = .01, *p* = .006). Months since diagnosis were not significant (*p* = .281). See Table 2 and Table 3.

However, no significant sex differences were found in fatigue severity, *F*(1, 334) =.85, *p* = .358, η^2^_*p*_ = .003. Neither covariate was significant (both *p*s ≥ .434). See Table 2 and Table 3.

#### 3.1.5. Emotional symptomatology.

Controlling for age and months since diagnosis, no significant sex differences were found in anxious symptomatology, *F*(1, 333) = 1.62, *p* = .204, η^2^_*p*_ = .005. Similarly, depressive symptomatology did not differ significantly by sex, *F*(1, 333) =.51, *p* = .477, η^2^_*p*_ = .002. Neither covariate was significantly associated with the outcome in any of the models (all *p*s ≥ .081). See Table 2 and Table 3.

### 3.2. Factors associated with health-related quality of life

#### 3.2.1. Exploratory associations with health-related quality of life by sex.

In women, dyspnoea severity (*B* = −4.893, *SE* = 1.39, *β* = −.212, *p* < .001), fatigue severity (*B* = −2.804, *SE* = .91, *β* = −.196, *p* = .002), anxious symptomatology (*B* = −.657, *SE* = .31, *β* = −.172, *p* = .034) and depressive symptomatology (*B* = −.810, *SE* = .27, *β* = −.238, *p* = .003) were negatively associated with HRQoL scores. In this model (Step 4), which included age, months since diagnosis, smoking status, physical activity, dyspnoea severity, fatigue severity, anxious symptomatology and depressive symptomatology, the regression model was significant (*F*(9, 235) = 15.49, *p* < .001), explaining 37% of the variance in HRQoL (adjusted *R*^2^ = .35, ∆*R*^2^ = .20, ∆*F*(4, 235) = 18.38, *p* < .001). See Table 4.

In men, months since diagnosis (*B* = .392, *SE* = .19, *β* = .213, *p* = .044) and fatigue severity (*B* = −5.377, *SE* = 1.45, *β* = −.432, *p* < .001) were significantly associated with HRQoL scores. A greater number of months since diagnosis was associated with higher HRQoL scores, whereas greater fatigue severity was associated with lower HRQoL scores. However, bootstrap analyses with 5,000 samples indicated that only fatigue severity remained significantly associated with HRQoL (*B* = −5.377, *SE* = 1.52, *p* = .001, 95% CI [−8.69, −2.63]). In this model (Step 4), the regression was significant (*F*(9, 64) = 5.58, *p* < .001), explaining 44% of the variance in HRQoL scores (adjusted *R*^2^ = .36, ∆*R*^2^ = .21, ∆*F*(4, 64) = 6.11, *p* < .001). See Table 5.

#### 3.2.2. Sex-by-predictor interaction analysis.

Hierarchical regression analysis of the full sample showed that the main effects in Step 4 were statistically significant, *F*(10, 308) = 17.920, *p* < .001, and explained 36.8% of the variance in HRQoL scores (adjusted *R*^2^ = .347). In this model, greater dyspnoea severity (*B* = −4.182, *SE* = 1.190, *β* = −.187, *p* < .001), fatigue severity (*B* = −3.361, *SE* = .757, *β* = −.242, *p* < .001), and depressive symptomatology (*B* = −.864, *SE* = .242, *β* = −.249, *p* < .001) were independently associated with lower HRQoL. Physical activity, smoking status, anxiety symptoms, age, months since diagnosis, and sex were not independently associated with HRQoL in the main-effects model (all *p*s ≥ .129) (Table 6).

Adding the seven sex-by-predictor interaction terms in Step 5 did not significantly improve model fit, Δ*R*^2^ = .015, Δ*F*(7, 301) = 1.069, *p* = .384. None of the individual sex-by-predictor interaction terms reached statistical significance (all *p*s ≥ .117). Fatigue remained significantly associated with lower HRQoL in the final model (*B* = −5.423, *p* < .001). However, as main-effect coefficients in this model are conditional on the reference sex category, the interaction block was used primarily to assess sex moderation. Thus, there was no statistically detectable evidence that the associations between smoking status, physical activity, dyspnoea, fatigue, anxiety, or depressive symptoms and HRQoL differed by sex (Table 6).

## 4. Discussion

The present study examined sex differences in long COVID symptomatology, HRQoL, smoking behaviour, physical activity, dyspnoea severity, fatigue severity, and emotional symptomatology in individuals with long COVID. In addition, sex-specific factors associated with HRQoL were analysed using hierarchical regression models conducted separately for women and men. Finally, an exploratory moderation analysis was conducted in the full sample to examine whether the associations between the examined factors and HRQoL differed by sex. Several relevant findings emerged. In the exploratory analyses of symptom prevalence, based on patient-reported data from the ad hoc symptom checklist, women reported a higher prevalence of several symptoms, with a greater prevalence of palpitations, headaches, cognitive complaints, gastrointestinal symptoms, and arthralgia. In addition, women reported lower HRQoL scores than men, and a higher likelihood of experiencing more severe dyspnoea. No sex differences were observed in smoking behaviour, physical activity, fatigue severity, or anxiety and depressive symptoms. In exploratory sex-stratified analyses, dyspnoea severity, fatigue severity, and anxiety and depressive symptoms were associated with poorer HRQoL in women, whereas fatigue severity was the only factor significantly associated with HRQoL in men. However, the interaction analysis did not provide statistically significant evidence that these associations differed by sex.

### 4.1. Distribution of symptoms in women and men

Although women and men with long COVID share many common symptoms, our results indicate that long COVID does not affect both sexes uniformly, with several symptoms being significantly more prevalent in women than in men. These findings are consistent with previous literature identifying female sex as a risk factor for the development of post-acute sequelae following COVID-19 infection [6]. In particular, women in our sample reported a higher prevalence of cognitive symptoms, including brain fog and memory problems, in line with previous studies documenting a higher frequency of cognitive complaints such as brain fog and concentration difficulties among women with long COVID [6,7]. Likewise, our findings suggest that women may experience a higher burden of pain-related symptoms, such as headaches and arthralgia, compared with men, consistent with previous findings reporting worse scores in the pain/discomfort domain of the EQ-5D among women, regardless of the severity of the initial infection [36]. In addition, a higher prevalence of gastrointestinal symptoms, specifically diarrhoea, was observed among women compared with men, extending previous observations of sex differences in gastrointestinal manifestations reported during the acute phase of COVID-19 to the long COVID context [37].

Taken together, these sex-related differences in symptom burden may be partly explained by biological mechanisms related to immune regulation. Sex hormone–and X chromosome–related modulation of immune responses has been proposed to influence COVID-19 manifestations across sexes, potentially conferring protection against severe acute disease while simultaneously favouring immune persistence or dysregulation, thereby increasing vulnerability to long COVID symptoms in women [6,38].

### 4.2. Sex differences in HRQoL, physical activity patterns and dyspnoea severity

In the present study, women with long COVID reported significantly lower HRQoL than men, as measured by the EQ-5D VAS, even after controlling for age and time since diagnosis. This finding suggests that female sex is intrinsically associated with poorer perceived HRQoL in the context of long COVID. The result aligns with the findings of a previous study in the literature [39].

The poorer HRQoL observed in women may have a multifactorial origin. In our cohort, women presented a higher symptom burden than men, which may be contributing to greater functional limitations and poorer perceived quality of life. However, studies conducted in chronic diseases suggest that differences in quality of life may also be explained, at least in part, by differences in illness perception and coping [40]. Women tend to adopt a more autonomous and proactive role in the management of their disease [41] and use healthcare services more frequently [42]. Men, on the other hand, appear to rely more on external support [41], tend to report fewer physical and mental symptoms, and delay seeking healthcare [43]. In addition, psychosocial factors may also contribute to these differences. Women [43] often assume a greater burden of caregiving responsibilities and may experience a greater emotional burden [44], which could amplify the impact of the disease on daily life and, consequently, on HRQoL.

Physical activity may also be relevant when considering sex differences in HRQoL. In our sample, women showed a pattern of lower physical activity levels than men, although this difference was not statistically conclusive in the robust analysis. Lower physical activity has been associated with poorer HRQoL in individuals with long COVID [45]. Reduced engagement in physical activity may, in turn, reflect limitations in functional capacity related to persistent symptoms.

One potential explanation for these limitations in functional capacity is the greater severity of dyspnoea observed among women in our sample. In line with this, a previous study has reported a higher likelihood of persistent dyspnoea among women with long COVID [46]. Dyspnoea is a clinically relevant and functionally limiting symptom that has been associated with restrictions in activities of daily living and functional disability in long COVID [47]. Accordingly, greater dyspnoea severity may contribute to reduced exercise tolerance [23], which could potentially affect physical activity levels and, in turn, HRQoL.

Interestingly, no sex differences were observed in smoking behaviour, fatigue severity, or anxiety and depressive symptoms. Given that smoking behaviour is highly context-dependent and varies by country, sex, and age [48], this might contribute to the heterogeneity observed across long COVID studies. While Bai et al. [49] reported higher rates of active smoking among men and Leone et al. [50] found differences mainly between never-smokers and ex-smokers in Italian patients, our study showed no sex differences in smoking status or smoking exposure expressed as pack-years.

Regarding fatigue severity, the existing literature is not consistent as to whether sex differences are present. On the one hand, the systematic review by Gorenshtein et al. [6] reported that women with long COVID experience fatigue more frequently than men; however, unlike the present study, this systematic review focused on fatigue prevalence rather than fatigue severity. In contrast, other studies examining fatigue severity using FSS have similarly found no significant differences between women and men with long COVID [51]. Together, these findings suggest that sex differences in fatigue may be more apparent at the level of symptom prevalence than in perceived severity.

A similar explanation may apply to the absence of sex differences observed in emotional symptomatology in our study. Differences in the assessment instruments used may partly account for these findings. The OASIS and ODSIS differ from other commonly used anxiety and depression measures in that they assess symptom severity dimensionally, integrating frequency, intensity, and functional interference over the past week [34]. With respect to depression, women with long COVID appear to show a higher prevalence of depressive symptoms than men [6]; however, this does not necessarily imply greater functional interference. On the other hand, the systematic review by Gorenshtein et al. [6] found no consistent sex differences in anxiety prevalence in long COVID and highlighted substantial heterogeneity across the available literature.

### 4.3. Exploratory associations with HRQoL in women and men

Although women reported a higher overall symptom burden in long COVID [36], the impact of symptoms on HRQoL is likely to depend not only on the number of symptoms but also on their functional relevance [52]. Previous research using multivariate models has identified fatigue and affective symptoms as key factors associated with HRQoL impairment in individuals with long COVID [53,54]. However, most of these findings have relied on aggregated analyses that adjust for sex, rather than explicitly examining whether the factors associated with HRQoL differ between women and men.

Given the documented sex differences in long COVID symptomatology [6,36], exploring sex-specific patterns of association with HRQoL represents an important and still underexplored research question. The sex-stratified exploratory analyses showed different within-sex patterns of association with HRQoL. Among women, poorer HRQoL was associated with a combination of physical and emotional factors. Specifically, greater dyspnoea severity, higher fatigue severity, and higher levels of anxious and depressive symptomatology were significantly associated with poorer HRQoL, highlighting the multifactorial nature of quality-of-life impairment in this group. In contrast, among men, fatigue severity was the only factor significantly associated with HRQoL. However, the analysis of the interaction between sex and predictor did not provide statistically significant evidence that these associations differed between women and men. Therefore, the sex-stratified findings should be interpreted as exploratory patterns rather than as evidence of confirmed sex differences in how dyspnoea, fatigue or emotional symptoms affect HRQoL.

Taken together, these results extend previous research by suggesting that, while fatigue and affective symptoms are important determinants of HRQoL in long COVID at the population level, their associations with HRQoL may differ between women and men. These findings are consistent with evidence indicating that HRQoL impairments in long COVID arise from the combined influence of physical symptoms and emotional distress, and that sex is independently associated with poorer HRQoL outcomes [54]. Moreover, evidence from other chronic conditions suggests that the relative contribution of these physical and emotional factors to quality of life may vary by sex, supporting the relevance of analysing HRQoL determinants separately in women and men [55].

### 4.4. Limitations, future directions and conclusion

Our study has several limitations. Firstly, participation was voluntary, which may have introduced selection bias, as individuals with more severe long COVID symptoms may have been less likely to accept enrolment. Consequently, the findings may not be fully generalisable to the entire long COVID population. Furthermore, the higher proportion of women in our sample may also have contributed to selection bias. In addition to female sex being identified as a risk factor for the development of post-acute sequelae following COVID-19 infection [6], women have been shown to adopt a more active role in managing their health and to use healthcare services more frequently than men [41,42]. As participants were recruited through healthcare services, women may therefore have been more likely to be identified and invited to participate. Consequently, the possibility of sex-related selection bias cannot be excluded and should be considered when interpreting the observed sex differences. Furthermore, the comparatively small male subgroup reduced the precision of the male-specific estimates. These findings should therefore be interpreted cautiously.

Secondly, socioeconomic status was not assessed in the present study. Recent evidence suggests that socioeconomic position may influence the relationship between long COVID and HRQoL [56]. Therefore, we cannot exclude the possibility that socioeconomic factors influenced the HRQoL outcomes observed in our cohort, and this should be considered when interpreting our findings. In addition, information on employment status, sick leave, domestic workload, and caregiving responsibilities was not collected. Although information on living arrangements was available (see Table 1), this variable does not allow us to determine whether participants had caregiving responsibilities or household-related burdens. Therefore, these unmeasured factors may have influenced, or been associated with, physical activity levels and HRQoL.

Thirdly, all variables were assessed using self-reported measures, potentially introducing reporting and common-method biases. In particular, physical activity was assessed using a single-item indicator referring to weekly hours devoted specifically to physical exercise. This measure did not capture the type, intensity, or context of exercise, nor did it capture daily fluctuations in activity. Smoking behaviour was assessed using self-reported smoking status (current smoker, ex-smoker, or never smoker), an approach commonly used in long COVID research [49,50]. Nevertheless, the absence of a standardised or objective assessment may have reduced the sensitivity to capture more nuanced smoking-related associations. To solve this limitation, future studies using ecological momentary assessment tools such as smartphones or smartwatches will improve the collection of this type of data in real time and in the natural environment of participants.

Fourthly, the wide variability in time since diagnosis introduces clinical heterogeneity which, although partially controlled for through statistical adjustment, cannot be fully eliminated. Taken together, these limitations suggest that the findings should be generalised with caution, particularly to populations with different demographic characteristics or healthcare settings.

Finally, while the present study examined sex differences in HRQoL and its associated factors in long COVID, future research should adopt longitudinal designs to explore sex-specific trajectories over time and to investigate the biological, psychological, and social mechanisms underlying these differences. In addition, sex-sensitive intervention studies may help identify more effective rehabilitation strategies to improve quality of life in women and men with long COVID.

In conclusion, the present findings indicate that women with long COVID experience a greater symptom burden, poorer health-related quality of life and greater dyspnoea severity than men. Moreover, exploratory sex-stratified analyses showed different within-sex patterns of association with HRQoL: among women, poorer HRQoL was associated with physical and emotional symptoms, whereas among men, fatigue severity was the only factor significantly associated with HRQoL.

However, the formal interaction analysis did not provide statistically significant evidence that these associations differed by sex. These results underscore the importance of adopting a sex-sensitive approach to the assessment of quality of life and symptom burden in long COVID and highlight the clinical relevance of identifying and addressing the symptoms with the greatest functional impact in women and men.

## References

[1] World Health Organization [WHO]. A clinical case definition of post COVID-19 condition by a Delphi consensus. 2021. [cited 2022 Sep 7]. Available from: https://www.who.int/publications/i/item/WHO-2019-nCoV-Post_COVID-19_condition-Clinical_case_definition-2021.1/10.1016/S1473-3099(21)00703-9 PMC8691845 34951953

[2] Davis HE, McCorkell L, Vogel JM, Topol EJ. Long COVID: Major findings, mechanisms and recommendations. Nat Rev Microbiol. 2023;21(3):133–46. doi: 10.1038/s41579-022-00846-2 36639608 PMC9839201

[3] Llana T, Mendez M, Zorzo C, Fidalgo C, Juan M-C, Mendez-Lopez M. Anosmia in COVID-19 could be associated with long-term deficits in the consolidation of procedural and verbal declarative memories. Front Neurosci. 2022;16:1082811. doi: 10.3389/fnins.2022.1082811 36570827 PMC9780694

[4] Llana T, Garces-Arilla S, Juan M-C, Mendez-Lopez M, Mendez M. An immersive virtual reality-based object-location memory task reveals spatial long-term memory alterations in Long-COVID. Behav Brain Res. 2024;471:115127. doi: 10.1016/j.bbr.2024.115127 38936427

[5] Writing Committee for the COMEBAC Study Group, Morin L, Savale L, Pham T, Colle R, Figueiredo S, et al. Four-month clinical status of a cohort of patients after hospitalization for COVID-19. JAMA. 2021;325(15):1525–34. doi: 10.1001/jama.2021.3331 33729425 PMC7970386

[6] Gorenshtein A, Leibovitch L, Liba T, Stern S, Stern Y. Gender disparities in neurological symptoms of long COVID: A systematic review and meta-analysis. Neuroepidemiology. 2025;59(4):426–40. doi: 10.1159/000540919 39159607 PMC12324708

[7] Román-Montes CM, Flores-Soto Y, Guaracha-Basañez GA, Tamez-Torres KM, Sifuentes-Osornio J, González-Lara MF, et al. Post-COVID-19 syndrome and quality of life impairment in severe COVID-19 Mexican patients. Front Public Health. 2023;11:1155951. doi: 10.3389/fpubh.2023.1155951 37255755 PMC10225709

[8] Malik P, Patel K, Pinto C, Jaiswal R, Tirupathi R, Pillai S, et al. Post-acute COVID-19 syndrome (PCS) and health-related quality of life (HRQoL)-A systematic review and meta-analysis. J Med Virol. 2022;94(1):253–62. doi: 10.1002/jmv.27309 34463956 PMC8662132

[9] Sirotiak Z, Lee D-C, Brellenthin AG. Associations between physical activity, long COVID symptom intensity, and perceived health among individuals with long COVID. Front Psychol. 2024;15:1498900. doi: 10.3389/fpsyg.2024.1498900 39507087 PMC11538967

[10] The WHOQOL Group. The World Health Organization Quality of Life Assessment (WHOQOL): Development and general psychometric properties. Soc Sci Med. 1998;46(12):1569–85. doi: 10.1016/s0277-9536(98)00009-4 9672396

[11] Amdal CD, Pe M, Falk RS, Piccinin C, Bottomley A, Arraras JI, et al. Health-related quality of life issues, including symptoms, in patients with active COVID-19 or post COVID-19; A systematic literature review. Qual Life Res. 2021;30(12):3367–81. doi: 10.1007/s11136-021-02908-z 34146226 PMC8214069

[12] Ware JE, Snow KK, Kosinski M, Gandek B. SF-36 Health Survey: Manual and Interpretation Guide. Boston, MA: The Health Institute; 1993.

[13] Chen K-Y, Li T, Gong F-H, Zhang J-S, Li X-K. Predictors of health-related quality of life and influencing factors for COVID-19 patients, a follow-up at one month. Front Psychiatry. 2020;11:668. doi: 10.3389/fpsyt.2020.00668 32733299 PMC7360857

[14] Brooks R. EuroQol: The current state of play. Health Policy. 1996;37:53–72. doi: 10.1016/0168-8510(96)00822-6 10158943

[15] Brazier JE, Rowen D, Lloyd A, Karimi M. Future directions in valuing benefits for estimating QALYs: Is time up for the EQ-5D? Value Health. 2019;22:62–8. doi: 10.1016/j.jval.2018.12.001 30661635

[16] Caamano E, Velasco L, Garcia MV, Asencio JM, Piñeiro P, Hortal J, et al. Prognostic factors for deterioration of quality of life one year after admission to ICU for severe SARS-COV2 infection. Qual Life Res. 2024;33(1):123–32. doi: 10.1007/s11136-023-03503-0 37615735

[17] Mastrorosa I, Del Duca G, Pinnetti C, Lorenzini P, Vergori A, Brita AC, et al. What is the impact of post-COVID-19 syndrome on health-related quality of life and associated factors: a cross-sectional analysis. Health Qual Life Outcomes. 2023;21(1):28. doi: 10.1186/s12955-023-02107-z 36949439 PMC10031164

[18] Nandasena HMRKG, Pathirathna ML, Atapattu AMMP, Prasanga PTS. Quality of life of COVID 19 patients after discharge: Systematic review. PLoS One. 2022;17(2):e0263941. doi: 10.1371/journal.pone.0263941 35171956 PMC8849513

[19] Gidey K, Niriayo YL, Asgedom SW, Lubetkin E. Health-related quality of life in COVID-19 patients: A systematic review and meta-analysis of EQ-5D studies. Health Qual Life Outcomes. 2025;23(1):97. doi: 10.1186/s12955-025-02421-8 41057923 PMC12506383

[20] Paul E, Fancourt D. Health behaviours the month prior to COVID-19 infection and the development of self-reported long COVID and specific long COVID symptoms: A longitudinal analysis of 1581 UK adults. BMC Public Health. 2022;22(1):1716. doi: 10.1186/s12889-022-14123-7 36085055 PMC9462889

[21] Joseph G, Margalit I, Weiss-Ottolenghi Y, Rubin C, Murad H, Gardner RC, et al. Persistence of long COVID symptoms two years after SARS-CoV-2 infection: A prospective longitudinal cohort study. Viruses. 2024;16(12):1955. doi: 10.3390/v16121955 39772261 PMC11680455

[22] Espejo-Paeres C, Núñez-Gil IJ, Estrada V, Fernández-Pérez C, Uribe-Heredia G, Cabré-Verdiell C, et al. Impact of smoking on COVID-19 outcomes: A HOPE Registry subanalysis. BMJ Nutr Prev Health. 2021;4(1):285–92. doi: 10.1136/bmjnph-2021-000269 34308137 PMC8214987

[23] Betschart M, Rezek S, Unger I, Ott N, Beyer S, Böni A, et al. One year follow-up of physical performance and quality of life in patients surviving COVID-19: A prospective cohort study. Swiss Med Wkly. 2021;151:w30072. doi: 10.4414/smw.2021.w30072 34751538

[24] Özkeskin M, Özden F, Karaman B, Ekmekçi Ö, Yüceyar N. The comparison of fatigue, sleep quality, physical activity, quality of life, and psychological status in multiple sclerosis patients with or without COVID-19. Mult Scler Relat Disord. 2021;55:103180. doi: 10.1016/j.msard.2021.103180 34352513 PMC8324500

[25] Tak CR. The health impact of long COVID: a cross-sectional examination of health-related quality of life, disability, and health status among individuals with self-reported post-acute sequelae of SARS CoV-2 infection at various points of recovery. J Patient Rep Outcomes. 2023;7(1):31. doi: 10.1186/s41687-023-00572-0 36943643 PMC10029785

[26] Tanguay P, Décary S, Lemaire-Paquette S, Léonard G, Piché A, Dubois M-F, et al. Trajectories of health-related quality of life and their predictors in adult COVID-19 survivors: A longitudinal analysis of the Biobanque Québécoise de la COVID-19 (BQC-19). Qual Life Res. 2023;32(9):2707–17. doi: 10.1007/s11136-023-03406-0 37004627 PMC10066943

[27] Gobierno de Aragón. Proceso asistencial para la atención a las personas con condición post COVID-19 en el ámbito de la atención primaria y criterios para su derivación a la atención hospitalaria. Zaragoza. 2021. Available from: https://www.aragon.es/documents/20127/90404045/proceso+asistencial++para+la+atención++a+los+pacientes+con+covid+persistente.pdf/64efb985-2dfe-1f4e-d106-5a95f9999855?t=1637584685096

[28] Badia X, Roset M, Montserrat S, Herdman M, Segura A. La versión española del EuroQol: Descripción y aplicaciones. Med Clin (Barc). 1999;112:79–86. 10618804

[29] Derrett S, Black J, Herbison GP. Outcome after injury-a systematic literature search of studies using the EQ-5D. J Trauma. 2009;67(4):883–90. doi: 10.1097/TA.0b013e3181ae6409 19820601

[30] Natori H, Kawayama T, Suetomo M, Kinoshita T, Matsuoka M, Matsunaga K, et al. Evaluation of the Modified Medical Research Council Dyspnea Scale for Predicting Hospitalization and Exacerbation in Japanese patients with chronic obstructive pulmonary disease. Intern Med. 2016;55(1):15–24. doi: 10.2169/internalmedicine.55.4490 26726080

[31] Learmonth YC, Dlugonski D, Pilutti LA, Sandroff BM, Klaren R, Motl RW. Psychometric properties of the Fatigue Severity Scale and the Modified Fatigue Impact Scale. J Neurol Sci. 2013;331(1–2):102–7. doi: 10.1016/j.jns.2013.05.023 23791482

[32] Duarte RE, Velasco E, Sánchez-Sosa JJ, Reyes-Lagunes LI. Validación psicométrica de la Escala de gravedad de fatiga en médicos residentes mexicanos. Educación Médica. 2019;20(1):28–36. doi: 10.1016/j.edumed.2017.11.006

[33] Norman SB, Cissell SH, Means-Christensen AJ, Stein MB. Development and validation of an Overall Anxiety Severity And Impairment Scale (OASIS). Depress Anxiety. 2006;23(4):245–9. doi: 10.1002/da.20182 16688739

[34] Osma J, Quilez-Orden A, Suso-Ribera C, Peris-Baquero O, Norman SB, Bentley KH, et al. Psychometric properties and validation of the Spanish versions of the overall anxiety and depression severity and impairment scales. J Affect Disord. 2019;252:9–18. doi: 10.1016/j.jad.2019.03.063 30953927

[35] Bentley KH, Gallagher MW, Carl JR, Barlow DH. Development and validation of the Overall Depression Severity and Impairment Scale. Psychol Assess. 2014;26:815–30. doi: 10.1037/a0036216 24708078

[36] Barreto APA, Barreto Filho MA, Duarte LC, Cerqueira-Silva T, Camelier A, Tavares NM, et al. Metabolic disorders and post-acute hospitalization in black/mixed-race patients with long COVID in Brazil: A cross-sectional analysis. PLoS One. 2022;17(10):e0276771. doi: 10.1371/journal.pone.0276771 36315558 PMC9621406

[37] Sierpiński R, Pinkas J, Jankowski M, Zgliczyński WS, Wierzba W, Gujski M, et al. Sex differences in the frequency of gastrointestinal symptoms and olfactory or taste disorders in 1942 nonhospitalized patients with coronavirus disease 2019 (COVID-19). Pol Arch Intern Med. 2020;130(6):501–5. doi: 10.20452/pamw.15414 32491298

[38] Tramontana F, Battisti S, Napoli N, Strollo R. Immuno-endocrinology of COVID-19: The key role of sex hormones. Front Endocrinol (Lausanne). 2021;12:726696. doi: 10.3389/fendo.2021.726696 34925228 PMC8675353

[39] Galanis P, Katsiroumpa A, Vraka I, Kosiara K, Siskou O, Konstantakopoulou O, et al. Post-COVID-19 syndrome and related dysautonomia: reduced quality of life, increased anxiety and manifestation of depressive symptoms: Evidence from Greece. Acta Med Litu. 2023;30(2):139–51. doi: 10.15388/Amed.2023.30.2.6 38516508 PMC10952430

[40] Del Core MA, Ahn J, Wukich DK, Liu GT, Lalli T, VanPelt MD, et al. Gender differences on SF-36 patient-reported outcomes of diabetic foot disease. Int J Low Extrem Wounds. 2018;17:87–93. doi: 10.1177/1534734618774664 29929411

[41] Llubes-Arrià L, Briones-Vozmediano E, Mateos JT, Sol-Cullere J, Gea-Sánchez M, Rubinat-Arnaldo E. Gender differences in self-management activation among patients with multiple chronic diseases: A qualitative study. Arch Public Health. 2025;83(1):200. doi: 10.1186/s13690-025-01686-1 40745663 PMC12315370

[42] Thompson AE, Anisimowicz Y, Miedema B, Hogg W, Wodchis WP, Aubrey-Bassler K. The influence of gender and other patient characteristics on health care-seeking behaviour: A QUALICOPC study. BMC Fam Pract. 2016;17:38. doi: 10.1186/s12875-016-0440-0 27036116 PMC4815064

[43] Courtenay W. Key determinants of the health and well-being of men and boys. Int J Mens Health. 2003;2(1):1–30. doi: 10.3149/jmh.0201.1

[44] Sanders JE, Carrothers E, Gamble O, Neeb D, Arundel MK. The cost of caregiving: The disproportionate and invisible impact of COVID-19 on women. Affilia. 2025;40(3):471–89. doi: 10.1177/08861099251317493 PMC12250837 40657570

[45] Chao T-C, Chiang S-L, Lai C-Y, Huang C-Y, Lee M-S, Lin C-H, et al. Association between physical activity amount and cardiorespiratory fitness, sleep quality, and health-related quality of life in patients with long COVID: A cross-sectional study. Arch Phys Med Rehabil. 2024;105(9):1673–81. doi: 10.1016/j.apmr.2024.04.010 38697594

[46] Duwel V, de Kort JML, Becker CM, Kock SM, Tromp GG, Busari JO. A cross-sectional study of the physical and mental well-being of long COVID patients in Aruba. Clin Med Res. 2023;21(2):69–78. doi: 10.3121/cmr.2023.1821 37407214 PMC10321723

[47] Fernández-de-Las-Peñas C, Palacios-Ceña D, Gómez-Mayordomo V, Palacios-Ceña M, Rodríguez-Jiménez J, de-la-Llave-Rincón AI, et al. Fatigue and dyspnoea as main persistent post-COVID-19 symptoms in previously hospitalized patients: Related functional limitations and disability. Respiration. 2022;101(2):132–41. doi: 10.1159/000518854 34569550 PMC8678253

[48] Liu H, Qi Q, Duan Y, Ma C, Zhou C. Sex and macroeconomic differences and trends in early attempts at cigarette smoking among adolescents: Findings from 147 countries. BMC Med. 2022;20(1):311. doi: 10.1186/s12916-022-02512-z 36131270 PMC9494856

[49] Bai F, Tomasoni D, Falcinella C, Barbanotti D, Castoldi R, Mulè G, et al. Female gender is associated with long COVID syndrome: A prospective cohort study. Clin Microbiol Infect. 2022;28(4):611.e9–611.e16. doi: 10.1016/j.cmi.2021.11.002 34763058 PMC8575536

[50] Leone V, Freuer D, Goßlau Y, Kirchberger I, Warm T, Hyhlik-Dürr A, et al. Symptom clusters in acute SARS-CoV-2 infection and long COVID fatigue in male and female outpatients. J Pers Med. 2024;14(6):602. doi: 10.3390/jpm14060602 38929823 PMC11205233

[51] Fietsam AC, Bryant AD, Rudroff T. Fatigue and perceived fatigability, not objective fatigability, are prevalent in people with post-COVID-19. Exp Brain Res. 2023;241(1):211–9. doi: 10.1007/s00221-022-06518-0 36462035 PMC9735153

[52] Kılınçarslan MG, Ocak Ö, Şahin EM. Health-related quality of life in long COVID-19 in context of symptom type. Ann Clin Anal Med. 2023;14:379–83. doi: 10.4328/acam.21508

[53] Maes M, Al-Rubaye HT, Almulla AF, Al-Hadrawi DS, Stoyanova K, Kubera M, et al. Lowered quality of life in long COVID is predicted by affective symptoms, chronic fatigue syndrome, inflammation and neuroimmunotoxic pathways. Int J Environ Res Public Health. 2022;19(16):10362. doi: 10.3390/ijerph191610362 36011997 PMC9408685

[54] Satar S, Şahin ME, Ergün P. Health related quality of life and its determinants in COVID-19 patients. Tuberk Toraks. 2023;71(3):250–60. doi: 10.5578/tt.20239706 37740628 PMC10795277

[55] Neto LO, Gromisch ES, Sloan J, Tyry T, Foley FW. Sex differences in predictors of illness intrusiveness in persons with multiple sclerosis. Qual Life Res. 2019;28(2):389–97. doi: 10.1007/s11136-018-2023-0 30334142

[56] Delpierre C, Lemogne C, Steichen O, Tebeka S, Makovski TT, Coste J. Influence of socioeconomic position on the relationship between long COVID and health-related quality of life: A nationwide random sampling survey in France in autumn 2022. Soc Sci Med. 2025;382:118368. doi: 10.1016/j.socscimed.2025.118368 40645034

